# Mild Stroke, Serious Problems: Limitations in Balance and Gait Capacity and the Impact on Fall Rate, and Physical Activity

**DOI:** 10.1177/15459683231207360

**Published:** 2023-10-25

**Authors:** Jolanda M. B. Roelofs, Sarah B. Zandvliet, Ingrid M. Schut, Anouk C. M. Huisinga, Alfred C. Schouten, Henk T. Hendricks, Digna de Kam, Leo A. M. Aerden, Johannes B. J. Bussmann, Alexander C. H. Geurts, Vivian Weerdesteyn

**Affiliations:** 1 Department of Rehabilitation, Donders Institute for Brain, Cognition and Behaviour, Radboud University Medical Center, Nijmegen, The Netherlands; 2 Department of Biomechanical Engineering, Delft University of Technology, Delft, The Netherlands; 3 Rehabilitation Centre Klimmendaal, Arnhem, The Netherlands; 4 Department of Biomechanical Engineering, Technical Medical Centre, University of Twente, Enschede, The Netherlands; 5 Department of Rehabilitation Medicine, Rijnstate Hospital Arnhem, Arnhem, The Netherlands; 6 Department of Neurology, Reinier de Graafgasthuis, Delft, The Netherlands; 7 Department of Rehabilitation Medicine, Erasmus MC University Medical Center Rotterdam, Rotterdam, The Netherlands; 8 Sint Maartenskliniek, Research, Nijmegen, The Netherlands

**Keywords:** stroke, stroke rehabilitation, transient ischemic attack, postural balance, gait, accidental falls

## Abstract

**Background:**

After mild stroke persistent balance limitations may occur, creating a risk factor for fear of falling, falls, and reduced activity levels. *Objective.* To investigate whether individuals in the chronic phase after mild stroke show balance and gait limitations, elevated fall risk, reduced balance confidence, and physical activity levels compared to healthy controls.

**Methods:**

An observational case-control study was performed. Main outcomes included the Mini-Balance Evaluation Systems Test (mini-BEST), Timed Up and Go (TUG), 10-m Walking Test (10-MWT), and 6-item version Activity-specific Balance Confidence (6-ABC) scale which were measured in 1 session. Objectively measured daily physical activity was measured for 7 consecutive days. Fall rate in daily life was recorded for 12 months. Individuals after a mild stroke were considered eligible when they: (1) sustained a transient ischemic attack or stroke longer than 6 months ago, resulting in motor and/or sensory loss in the contralesional leg at the time of stroke, (2) showed (near-) complete motor function, that is, ≥24 points on the Fugl-Meyer Assessment—Lower Extremity (range: 0-28).

**Results:**

Forty-seven healthy controls and 70 participants after mild stroke were included. Participants with stroke fell more than twice as often as healthy controls, had a 2 point lower median score on the mini-BEST, were 1.7 second slower on TUG, 0.6 km/h slower on the 10-MWT, and had a 12% lower 6-ABC score. Intensity for both total activity (8%) as well as walking activity (6%) was lower in the participants with stroke, while no differences were found in terms of duration.

**Conclusions:**

Individuals in the chronic phase after a mild stroke demonstrate persistent balance limitations and have an increased fall risk. Our results point at an unmet clinical need in this population.

## Introduction

Balance and gait limitations, resulting from impaired sensorimotor function, are an important contributor to disability and reduced quality of life post-stroke.^1-3^ Rehabilitation programs, therefore, often aim to improve balance and gait performance in individuals with profound sensorimotor impairments after stroke.^4^ However, in a substantial proportion of patients with initial sensorimotor impairments, these impairments recover relatively quickly after stroke, ultimately resulting in (near-) absent sensorimotor symptoms.^5^ Those individuals with a so-called “mild stroke” are often discharged home after a short hospital stay,^6,7^ yet they face challenges to resume their daily life activities after hospital discharge.^7^ These challenges in daily life might be related to persistent balance and gait limitations. Given the paucity of studies and practice guidelines regarding persisting motor impairments in individuals after mild stroke, more insights are required into the presence and potential consequences of balance and gait limitations in this specific group.

Balance and gait limitations after stroke are a key risk factor for accidental falls.^8^ Falling is a major problem in moderately to severely affected individuals after stroke, as their fall risk remains substantially elevated (2-10 fold increase) throughout all post-stroke phases as compared to the general population of older adults.^8^ Whether fall risk is also elevated in people with minor stroke has not yet been studied. This deserves further investigation, since falls can have serious physical and psychological consequences. Direct physical consequences of falls involve bruises, soft tissue injuries, and also more severe injuries such as fractures (0.6%-15% of the post-stroke falls).^8,9^ Moreover, falls after stroke have a substantial psychological impact, with 88% of individuals after stroke developing a subsequent fear of falling after the incident.^10,11^ Individuals after stroke who fell tend to become less physically active^11^ either due to fear of falling^12^ or because of physical injuries. In the long term, avoidance of activities contributes to restricted social participation as well as reduced cardiovascular fitness. As physical activity levels in community-dwelling individuals in the chronic phase after stroke are already low in terms of duration and intensity,^13^ further deconditioning may contribute to loss of independence. In sum, post-stroke balance and gait limitations and subsequent (risk of) falls have serious physical and psychological consequences that eventually result in limited physical activity. Fortunately, exercise appears to reduce fall rates after stroke,^14^ and well-designed exercise therapy is effective in reducing balance limitations in the chronic phase after stroke.^15^

While the literature on individuals with mild motor impairments after stroke is sparse, a few studies report impaired balance and gait capacity after mild stroke.^16-19^ However, it remains unclear what the impact of these balance and gait limitations is in terms of falls and their consequences. We therefore conducted an observational case–control study of individuals in the chronic phase after mild stroke in which balance and gait capacity, balance confidence, and physical activity were measured along with a 12-month follow-up period of self-reported falls. In this study, we addressed the following research questions: What is the fall rate in individuals in the chronic phase after mild stroke as compared to healthy individuals? Do individuals with mild stroke present with limitations in terms of balance and gait capacity and are they less physically active as compared to healthy individuals? And if so, do these differences also pertain to individuals after mild stroke without clinically-established lower limb impairments (ie, maximum Fugl-Meyer and Motricity Index scores)?

We hypothesized that, compared to healthy individuals, balance and gait capacity would be reduced in individuals in the chronic phase after mild stroke as measured with the mini-Balance Evaluation Systems Test (mini-BEST, Timed up and go [TUG], and 10-m walking test [10-MWT]). Furthermore, we hypothesized that participants with mild stroke would exhibit higher fall rates, greater fear of falling (as measured with the Activity-specific Balance Confidence [ABC] scale), as well as lower physical activity levels (as measured with a wearable activity monitor) than their healthy counterparts.

## Methods

### Design, Setting, and Participants

We conducted an observational case–control study using clinical assessments to evaluate balance and gait capacity, and balance confidence (ie, absence of fear of falling) which were measured in 1 session. Physical activity levels were measured for 7 consecutive days. Following these baseline assessments, falls in daily life were monitored during a 12-month follow-up period. Potential participants who had sustained a mild stroke were recruited from the outpatient departments of Rehabilitation and Neurology at several general hospitals in the Netherlands (Radboud University Medical Center in Nijmegen, Rijnstate Hospital in Arnhem, Reinier de Graaf Hospital in Delft), and through advertisements in local newspapers. Written informed consent was obtained from all participants. The study protocol was approved by the Medical Ethical Board of the region Arnhem-Nijmegen, CMO Arnhem-Nijmegen under number: NL53300.091.15 All procedures were conducted in accordance with the Declaration of Helsinki.

To be included, participants had to be in the chronic phase (>6 months) after a mild stroke. Mild stroke was defined as a unilateral supratentorial transient ischemic attack (TIA) or stroke that had resulted in motor and/or sensory loss in the contralesional leg at stroke onset, with (near-) complete clinical motor recovery of the paretic leg as defined by the Fugl-Meyer Assessment—Lower Extremity score ≥24 (FMA-LE; range: 0-28, motor selectivity items only) at study inclusion. Participants with stroke were excluded if they (1) suffered from neurological conditions other than stroke or musculoskeletal (eg, joint arthrosis or replacement) problems; (2) had severe cognitive problems (Montreal Cognitive Assessment [MoCA]^20^ <24); (3) used psychotropic medication; or (4) had persistent unilateral spatial neglect (Behavioral Inattention Test—Star Cancellation Test^21^ <44). A group of healthy control participants of the same age range as the individuals with stroke were recruited from the community who were assessed for compliance with the first 3 exclusion criteria.

An initial screening was conducted during an extensive telephone interview. Potentially eligible candidates were invited to the university hospital to complete further screening as well as the baseline assessment. At inclusion, the following demographics and clinical characteristics were registered for both study groups: sex, age, Body Mass Index (BMI), MoCA as a measure of cognition, Quantitative Vibration Threshold^22,23^ (QVT; range: 0-8) of the medial malleolus and first metatarsophalangeal joint as a measure of deep sensibility of the paretic leg (participants with mild stroke) or the average of both legs (healthy controls), and Functional Ambulation Categories^24^ (FAC; range: 0-5) as a measure of ambulation capacity. For the participants with mild stroke, we additionally registered duration of initial hospital admission. An admission of 3 or less days was considered a short stay. Next to that location of discharge; prescription of home-based physiotherapy; time since stroke; type of stroke; affected body side; FMA-LE without coordination items, as a measure of motor function of the paretic leg; and the Motricity Index—Lower Extremity score (MI-LE; range: 0-100) as a measure of muscle strength of the paretic leg, were registered.

### Outcome Measures

#### Balance and Gait Capacity and Balance Confidence

At baseline, participants performed 3 clinical balance and gait capacity tests. Balance capacity was defined as the act of maintaining, achieving, or restoring a state of control during standing, measured in a standardized environment.^25^ The mini-BEST (0-28 points) was used to quantify balance capacity. The mini-BEST involves an assessment of 4 balance subdomains (anticipatory balance, reactive balance, sensory orientation, and dynamic balance during gait). The ceiling effect of the mini-BEST is limited, which allows for the detection of balance limitations in high-functioning individuals.^26^

Gait capacity was quantified by the TUG and 10-MWT and defined as moving along a surface on foot, where 1 foot is always on the ground, measured in a standardized environment.^27^ The TUG was specifically used to quantify functional mobility,^28^ and the 10-MWT was used to asses comfortable walking speed, as 2 different aspects of gait capacity.^19,28^

Participant’s balance confidence during common everyday activities was assessed with the 6-item short version of the ABC scale (6-ABC; range: 0%-100%),^29^ with 100% representing full balance confidence.

#### Falls

Falls in daily life were prospectively registered up until 12 months after the baseline assessments using monthly fall calendars.^30^ Each fall calendar was provided with a stamped and addressed envelope. Participants who did not return the fall calendar within 2 weeks after the end of each month were called to determine if any falls had occurred. A fall was defined as an unexpected event which resulted in body contact with the ground, floor or a lower level surface.^31^ Falls during sports or caused by a high-energy external force (eg, a collision) or loss of consciousness were excluded. All participants with one or multiple reported falls were considered fallers. The primary outcome for falls was fall rate (number of falls per person-year). As secondary outcomes, we used the proportion of fallers and the number of injurious falls during the 12-month follow-up.

#### Daily Physical Activity

Daily physical activity levels were registered for 7 days, 24 hours a day, directly following baseline assessment using the professional version of the Activ8 Physical Activity Monitor (Activ8; Remedy Distribution Ltd., Valkenswaard, The Netherlands). The Activ8 is a small (30 mm × 32 mm × 10 mm) and lightweight (20 g) one-sensor device with a triaxial accelerometer and is validated in patients with stroke.^32^ The sensor was attached with a waterproof Tegaderm^TM^ skin tape to the thigh of the non-paretic (mild stroke) or dominant (healthy control) leg. Participants received no feedback about their daily physical activity levels.

From the Activ8 accelerometer recordings, the duration and intensity (expressed in movement counts) of 6 categories of body postures and activities were identified (lying, sitting, standing, walking, cycling, and running), and summarized over 30-second epochs. In our analyses, we focused on the merged activities (ie, walking, cycling, and running) and, in addition, on the walking activity separately. The duration (in minutes) of these 2 activity categories was directly calculated from the Activ8 output. Intensity (counts/minutes) of both categories was computed by dividing the summed movement counts of each of the 2 activity categories by their duration.

### Required Sample Size

We anticipated a fall rate (falls per person-year) of 0.65 in healthy controls and expected this rate to double in individuals after a mild stroke.^8,33^ Assuming a Poisson distribution of falls data, a total sample of 110 participants was required to show a difference in fall rates between individuals after mild stroke and healthy controls with α = .05 and a power of 0.95. We choose to do this with a 3 to 2 ratio, requiring 66 individuals after mild stroke and 44 healthy controls.

### Statistical Analyses

Participant characteristics were compared between participants with mild stroke and healthy controls using an independent samples *t*-test or Mann–Whitney *U* test for continuous and ordinal variables, respectively. A Mann–Whitney *U* test was also used to compare balance confidence between groups. To compare balance and gait capacity outcomes and daily physical activity levels between groups, we conducted analysis of covariance with age as a covariate. We adjusted for age in our analyses as it is known from literature that balance and gait capacity declines with age.^34^ Between-group comparisons of fall rates were performed using Poisson regression with number of falls as dependent variable and group (mild stroke/healthy control) as independent variable. All participants with ≥6 months self-reported fall registration were included in the analyses. We conducted a Chi-square analysis to compare the proportion of fallers between groups.

Furthermore, we repeated the above-mentioned analyses for a subgroup of participants with complete clinical motor recovery of the paretic leg at inclusion, that is, FMA-LE = 28 and MI-LE = 100, further referred to as the *full recoverers*. All statistical analyses were performed in SPSS (version 27.0). *P*-values <.05 were considered statistically significant.

## Results

**Figure 1.**
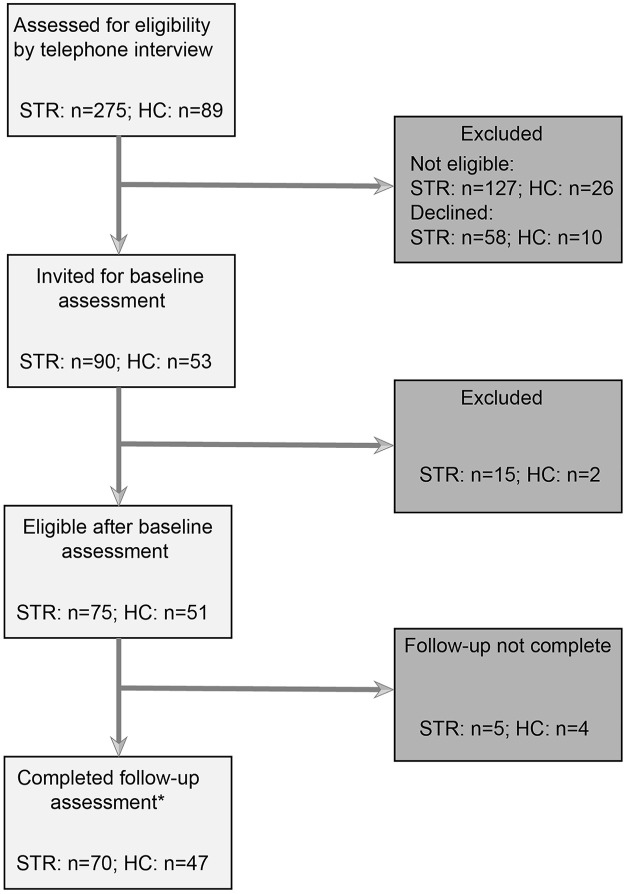
Flow of participants. Abbreviations: STR, mild stroke participant; HC, healthy control participant.

Between August 2016 and February 2019, 275 community-dwelling persons in the chronic phase (>6 months) after stroke and 89 healthy controls were assessed for eligibility, of whom 75 participants with mild stroke and 51 healthy controls were included (Figure 1). Of these participants, 70 participants with stroke and 47 controls were included in the data analyses, as they completed the minimally required 6 months self-reported fall registration. Within this group of 117 subjects were several missing observations; the 10-MWT in 1 participant with mild stroke, physical activity data of 4 participants with mild stroke and 6 controls, an incorrectly or not filled out ABC questionnaire in 12 participants with mild stroke and 3 controls. All sub-analyses were conducted using the available data of the 117 subjects. No imputation procedure was used.

Table 1 shows the participant characteristics. Following their acute stroke, 93% of the participants with mild stroke were discharged home following a short (median ≤3 days) hospital admission, and 47% subsequently received home-based physiotherapy. Thirty-eight participants with mild stroke (56%) had a maximum score on the FMA-LE and MI-LE and were classified as full recoverers. All participants were able to stand and walk independently on an irregular surface without supervision (FAC 5). No significant differences in sex and age were found between groups. BMI was higher for participants with mild stroke compared to healthy controls, while they scored slightly lower on cognition (MoCA) and deep sensibility, as tested on the metatarsophalangeal joint (QVT).

**Table 1.** Participants’ Characteristics.

Characteristics	All participants after mild stroke (n = 70)	Full recoverers after mild stroke (n = 38)^a^	Healthy control participants (n = 47)
Sex (male/female); n	43/27	23/15	23/24
Age (years); mean (range)	65.2 (43-85)	62.4 (43-74)	63.7 (42-82)
Body mass index; mean (range)	26.9 (19.5-39.0)^b^	26.6 (20.5-39.0)	24.9 (19.5-33.5)
MoCA; median (range)	27 (24-30)^c^	27 (24-30)	29 (24-30)
QVT-affected medial malleolus; median (range)	5.0 (0-8)	5.3 (0-8)	5.8 (2.5-8)
QVT-affected first MTP; median (range)	5.0 (0-8)^b^	5.1 (0-8)	5.6 (0-8)
FAC; % FAC 5	100	100	100
Duration of hospital admission; median (range) (≤3 days/>3 days/unknown); n	3.0 (0-14) 38/27/5	3.0 (0-14) 21/13/4	
Location of discharge (home/inpatient rehabilitation center); n	65/5	35/3	
Home-based physiotherapy (yes/no); n	33/37	13/25	
Time since stroke (months); median (range)	19.5 (5-183)	22.5 (5-183)	
Type of stroke (ischemic/hemorrhagic/unknown); n	63/5/2	36/2/-	
Affected body side (left/right); n	35/35	19/19	
FMA-LE; median (range)	28 (24-28)	28	
MI-LE; median (range)	100 (63-100)	100	

Abbreviations: MoCA, Montreal Cognitive Assessment (range: 0-30); QVT, Quantitative Vibration Threshold (range: 0-8); MTP, metatarsophalangeal joint; FAC, Functional Ambulation Categories (range: 0-5); FMA-LE, Fugl-Meyer Assessment—Lower Extremity (range: 0-28); MI-LE, Motricity Index—Lower Extremity (range: 0-100).

a Subgroup of mild stroke participants with complete motor recovery of the paretic leg (ie, FMA-LE = 28 and MI-LE = 100).

b *P* < .01 for comparison with controls.

c *P* < .05 for comparison with controls.

### Balance, Gait, and Balance Confidence

**Figure 2.**
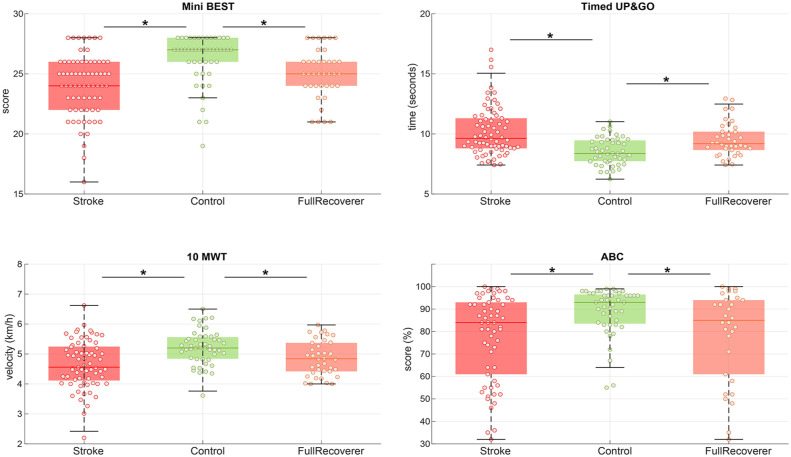
Balance and gait performance. Balance and gait scores for mild stroke participants versus controls. The full recoverers subgroup included participants with a mild stroke with a maximum score on the lower extremity scores of the Motricity Index and Fugl-Meyer assessment without coordination items. Individual datapoint are displayed with a circle, the colored line indicates the median and the surrounding box shows the interquartile range (IQR). The black error bars indicate the 25th percentile −1.5*IQR and 75th percentile −1.5*IQR, * = between group comparison showed a significant difference with a *P* < .05.

Participants in the mild stroke group exhibited poorer balance and gait scores than controls (mini-BEST score: median difference of 2 points, *P* < .01; TUG: median difference of 1.7 seconds, *P* < .01; 10-MWT: median difference of 0.6 km/h, *P* < .01; Figure 2). Lower balance confidence was found in the mild stroke group with a *median difference* of 12%, *P* < .01 on the ABC-score. Significantly lower scores in terms of balance and gait capacity as well as balance confidence were also observed for the full recoverers on the mini-BEST, TUG, and 10-MWT and ABC-score as compared to the healthy controls, see Figure 2 and Table 2.

**Table 2.** Results of Statistical Analyses.

Outcomes	All participants after mild stroke (n = 70)	Full recoverers after mild stroke (n = 38)^a^	Healthy control participants (n = 47)	Healthy vs all stroke participants	Healthy vs full recoverers
*mini-BEST* *median in points (IQR)*	24 (4)	25 (2)	26 (2)	*F* (1,114) = 24.1, B: −1.98, CI: −2.78 to −1.18, *P* < .01	*F* (1,82) = 7.2, B: −1.33, CI: −2.18 to −0.48, *P* < .01
*Timed Up & Go test* *Mean in seconds (IQR)*	10.20s (2.55)	9.53s (1.67)	8.54s (1.77)	*F* (1,114) = 25.6, B: 1.54, CI: 0.94, −2.15, *P* < .01	*F* (1,82) = 15.2, B: 1.04, CI: 0.51 to 1.57, *P* < .01
*10* *m walk test* *Mean in kilometer per hour (IQR)*	4.66 km/h (1.16)	4.86 km/h (0.96)	5.22 km/h (0.75)	*F* (1,113) = 14.8, B: −0.52, CI: −0.77 to −0.28, *P* < .01	*F* (1,82) = 10.4, B: −0.39, CI: −0.63 to −0.15, *P* < .01
6-item Activity-specific Balance Confidence scale *median in percentage (IQR)*	77% (33)	79% (34)	89% (14)	*U* = 801.5, *P* < .01	*U* = 479.0, *P* = .046
Fall events *Total number and falls per person-year*	n = 68, 0.97 per-y	n = 31, 0.82 per-y	n = 20, 0.43 per-y	RR = 2.3, CI: 1.39 to 3.76, *P* < .01	RR = 1.9, CI:1.09 to 3.36, *P* = .02
Fallers^b^ *Total number and percentage of group (ie, absolute fall risk)*	n = 33, 47%	n = 17, 45%	n = 16, 34%	χ^2^ = 1.98, *P* = .16	χ^2^ = 1.01, *P* = .31
Total duration of physical activity *Mean in minutes per day (IQR)*	169 min/d (79)	180 min/d (66)	178 min/d (65)	*F* (1,104) = 0.34, B: −5.74, CI: −25.24 to 13.76, *P* = .56	*F* (1,75) = 0.01, B: 1.10, CI: −19.77 to 21.98, *P* = .92
Total duration of walking *Mean in minutes per day (IQR)*	150 min/d (59)	161 min/d (204)	153 min/d (62)	*F* (1,104) = 0.00, B: 0.12, CI: −17.95 to 18.19, *P* = .99	*F* (1,75) = 0.61, B: 7.83, CI: −12.14 to 27.81, *P* = .44
Total intensity of physical activity *Mean in counts per minute (IQR)*	1494 c/min (255)	1540 c/min (276)	1609 c/min (236)	*F* (1,104) = 5.98, B: −99.87, CI: −180.84 to −18.9, *P* = .02	*F* (1,75) = 2.34, B: −76.22, CI: −175.58 to 23.14, *P* = .13
Total intensity of walking *Mean in counts per minute (IQR)*	1439 c/min (250)	1472 c/min (272)	1520 c/min (196)	F (1,104) = 4.38, B: −68.06, CI: −132.51 to −3.60, *P* = .04	F (1,75) = 2.02, B: −53.54, CI: −128.52 to 21.44, *P* = .16

Results of the statistical analyses conducted between mild stroke participants, healthy controls as well as a sub-analysis in which full recoverers were compared to healthy control participants. Between-group comparisons of fall rates were performed using Poisson regression giving relative risk (RR) as outcome and a Chi-square analysis was used to compare the proportion of fallers between groups.

a Subgroup of mild stroke participants with complete motor recovery of the paretic leg (ie, Fugl–Meyer Assessment–Lower Extremity = 28 and Motricity Index–Lower Extremity = 100).

b Participants were considered fallers when at least one fall event was reported.

**Figure 3.**
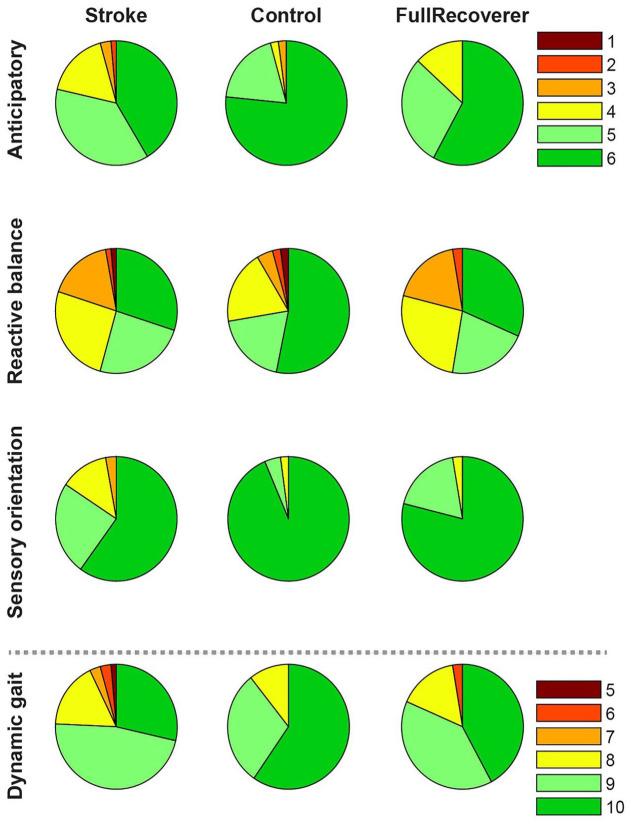
Mini-BEST sub-scores. Mini-BEST sub-scores for mild stroke participants vs. controls. The full recoverer subgroup included participants with a maximum score on the lower extremity scores of the Motricity Index and Fugl-Meyer assessment without coordination items. The pie charts represent the percentage of individuals with a given score on the Mini-Best subdomain.

Analysis of the mini-BEST sub-scores revealed that the mild stroke group performed worse than controls on all 4 domains (Figure 3). Compared to controls, the full recoverers exhibited poorer capacity on anticipatory and reactive balance, but not on the sensory and dynamic gait subscores.

### Falls

Participants with mild stroke had a 2.3 times higher relative risk of falling as compared to healthy controls (0.97 falls per person-year (per-year) vs 0.43 per-year, *P* < .01). Although full recoverers tended to fall less often than the total mild stroke group, they also exhibited a 1.9 times higher relative risk than controls (0.82 per-year vs 0.43 per-year *P* = .02). The percentage of fallers seemed higher in the mild stroke group (47%) compared to controls (34%), but did not reach statistical significance (see also Table 2). About half of the falls (ie, 54% in the mild stroke and 50% in the control group) resulted in mild injuries like cuts, bruises, pain, and/or joint sprains. Severe injuries (ie, dislocated joint and fracture) were rare with only 2 cases in the mild stroke group and 1 case in the control group.

### Daily Physical Activity

**Figure 4.**
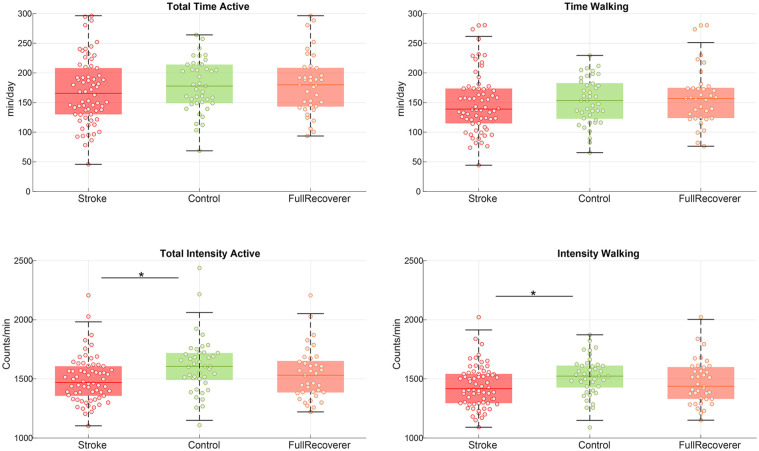
Physical activity. Physical activity for mild stroke participants versus controls. The full recoverer subgroup included participants with a maximum score on the lower extremity scores of the Motricity Index and Fugl-Meyer assessment without coordination items. Individual datapoint are displayed with a circle, the colored line indicates the median and the surrounding box shows the interquartile range (IQR). The black error bars indicate the 25th percentile −1.5*IQR and 75th percentile −1.5*IQR, * = between group comparison showed a significant difference with a *P* < .05.

Total duration of physical activity as well as the duration of walking were not different between participants after mild stroke and controls, see Figure 4. On the other hand, the intensity of activities in participants’ with mild stroke was lower for both total activities (8% lower, *P* = .02) as well as walking (6% lower, *P* = .04). Note that both the intensity and duration of walking closely followed the pattern of total activities (Figure 4), suggesting that the main type of physical activity was walking. The intensity of physical activity levels for the full recoverers tended to be closer to level of the control group. Between-group comparisons were no longer significant when only full recoverers were considered, see Table 2. The outcomes of the uncorrected statistical models can be found in Supplemental Table 1.

## Discussion

We investigated the impact of a mild stroke on balance and gait capacity, daily life falls, balance confidence and physical activity levels in 70 individuals in the chronic phase after mild stroke. Participants with a mild stroke performed worse in terms of balance and gait capacity compared to healthy controls. Remarkably, this was also the case for participants with full motor recovery of leg function, as defined by attaining maximum scores on two commonly used clinical tests of lower-extremity motor function. Next to that, participants with a mild stroke fell twice as often as healthy controls, exhibited poorer balance confidence and performed physical activities at a lower intensity.

### Individuals After Mild Stroke Exhibit Limitations Across Different Domains of Functional Balance and Gait Capacity

This study importantly adds to the sparse reports of balance and gait limitations after mild stroke in three ways. First, a careful selection of participants in the current study provided further insight in the widespread presence of balance and gait limitations in participants with mild stroke.^18,19^ Indeed, participants with severe cognitive impairments were excluded from our study, thereby reducing the likelihood that balance and gait capacity after mild stroke might have been affected by pre-existing micro-lesions or white matter disease. Second, a sub-analysis on full recoverers was performed, confirming that balance and gait limitations persisted in this seemingly well-recovered group of patients.^16^ Third, by monitoring fall events for one year, we showed the potential impact of these residual balance limitations after mild stroke.

### Fall Risk Is Substantially Elevated After Mild Stroke

With our longitudinal measurements we showed that there is an elevated risk of falling in daily life, which may well be linked to the observed persistent balance and gait limitations in individuals after mild stroke. The observed fall rate and the proportion of fallers after mild stroke in the current study is within the ranges that were previously reported for more severely affected individuals.^35,36^ This finding may be explained by the relatively high level of physical activity of our participants, which was comparable to that of healthy controls in terms of total activity duration and duration of walking. As such, our participants with mild stroke had a larger exposure to risky situations compared to more affected individuals after stroke that tend to spend less time physically active.^13^ In our study population, the reported falls generally did not have serious physical consequences, since no or relatively “mild” injuries were reported as a result of 97% of the falls. Yet, a history of falls is a strong predictor for future falls,^35^ causing an incremental risk of fall-related injuries.

### Reduced Balance Confidence After a Mild Stroke

While balance confidence in our study population seemed to be higher than in individuals with moderate to severe stroke,^37,38^ the median ABC score was still 9% lower than in healthy controls. Reduced balance confidence is a common consequence of falling.^10,11^ After mild stroke balance confidence deserves attention, since it may result in avoidance of physical activities by certain individuals. After a stroke poor balance confidence has been reported as a barrier for an active lifestyle^12^ and as one of the main factors that predicts physical inactivity 1 year after stroke.^39^ Moreover, reduced balance confidence is an independent determinant of the number of steps per day in individuals after stroke.^40^ In our cohort of individuals after mild stroke, reduced balance confidence was, on group level, paralleled by a lower intensity (rather than duration) of physical activity, which may reflect more cautious behavior. Avoidance of higher intensity physical activities after mild stroke may ultimately result in further deterioration of balance and gait performance, thereby increasing the risk of falling even more. Altogether, our results suggest that even though avoidance of physical activity does not seem to be an issue at this time point, balance confidence could give insights into a potential risk of avoidance in the future.

### Intensity, But Not Duration, of Physical Activity Is Reduced After Mild Stroke

Although no between-group differences in total time of physical activity were found, the median intensity of physical activity was 8% lower in our participants with a mild stroke compared to healthy control participants. As most of the total amount of physical activity involved walking, we focus our interpretation on walking intensity, which is closely linked to ambulatory walking speed.^41^ We found walking intensity to be reduced by 6% in the mild stroke group, which is comparable to the reduction found in comfortable walking speed as measured in the laboratory setting with the 10-MWT. The lower walking intensity in the mild stroke group could be the consequence of their leg motor function impairment (ie, lower scores on the FM-LE), poorer balance capacity (ie, lower scores on mini-BEST), lower balance confidence or lower cardiovascular capacity. It is likely that the observed reduction is caused by a combination of these factors, which cannot be distinguished from each other within this current study. In contrast, walking intensity was not significantly reduced when only considering the full recovers as compared to the health controls, while we did find a difference on the 10-MWT in this subgroup. These results hint at the possibility that even small reductions in motor function of the leg contribute to a lower walking intensity in daily life.

For individuals after stroke, being physically active at sufficiently high cardiovascular intensity is of particular importance for reducing cardiovascular risk factors.^42^ As people after mild stroke already have an increased cardiovascular risk profile^43^ (as indicated by their BMI-values in Table 1), enhancing the intensity of walking activity in daily life may be beneficial.

It should be mentioned, though, that walking, being a relatively low intensity exercise for healthy individuals, is known to impose greater cardiovascular demands on people after moderate to severe stroke due to a less efficient walking pattern requiring a higher energy expenditure.^44^ This may also be true for individuals after mild stroke who have some residual leg motor function impairments. The relationship between walking speed and cardiovascular intensity after stroke warrants further investigation, yet can be expected to be non-linear, and, among other factors, influenced by motor function of the paretic leg.

Taken together, the factors that contribute to lower intensity of physical activity intensity should be carefully considered for each individual after mild stroke to enhance secondary prevention of cardiovascular accidents, optimize participation in physical activities and decrease fall risk.

### Limitations

The current study has some limitations that may impact the interpretation and/or external validity of our results. First, it was not possible to determine the participants’ level of functioning before the onset stroke. Hence, it is possible that participants in the mild stroke group already performed worse than healthy controls stroke onset. Yet, it seems unlikely that the observed poorer functional capacity at group level of 70 individuals after mild stroke (Figure 2) is merely the result of pre-existing impairments in some individuals. Different from previous studies,^18,19^ we carefully selected participants with a clinically defined supratentorial stroke, thereby minimizing the possibility of cerebellar or brainstem contributions to the observed balance and gait limitations. However, we were unable to collect imaging data for all participants to fully exclude the possibility of additional lesions in the cerebellar or brainstem.

An important finding was that even individuals in the chronic phase after mild stroke were more prone to falling than age-matched healthy controls. Ideally, we would have collected more specific information about fall circumstances and precipitating factors to gain insight into the aetiology of falls in this population and to identify specific targets for intervention. Another limitation is that our current study sample was not large enough to study relationships between balance and gait capacity and reported falls, because falls are relatively occasional events with a multifactorial aetiology. Therefore, the determinants for falls in people with mild stroke need to be established in future large-scale longitudinal studies.

We used a definition of mild stroke based on clinically absent or limited residual motor impairments in the paretic leg (as determined by a score ≥24 on the FMA-LE) at study inclusion. This definition is in line with the definition of mild stroke used by Hu et al. 2017.^45^ While in theory it is possible that using this definition led us to include individuals who suffered from moderate or severe stroke in the acute phase but showed good recovery 6 months later, this possibility seems unlikely given the fact that 93% of the mild stroke participants in the current study were discharged home following a short (median ≤3 days) hospital stay. As we included our participants at least 6 months after stroke onset, it was not possible to categorize them as minor stroke based on their acute phase situation.^46,47^ We therefore chose to use the term “mild stroke” in our paper, although it is likely that the vast majority of our participants could have been classified as individuals with a minor stroke according to severity criteria at hospital admission.^46,47^

In this study we used the mini-BEST as a measure of balance capacity, because it has a smaller ceiling effect than the Berg Balance Scale.^48,49^ This was exemplified by the observed differences between healthy participants and individuals with mild stroke, even those who showed full leg motor recovery. Although we acknowledge that the median group differences of 2 points did not exceed the reported minimal clinically important change (MIC) values (4 points), it must be mentioned that 30% of our mild stroke participants deviated less than 4 points from the maximum score of 28 points. This seems to suggest that a ceiling effect may still have been present. Hence, the clinical utility of the mini-BEST may be limited for evaluating (changes in) balance and gait capacity in people with mild stroke. It is an interesting question for future research whether instrumented balance assessments (eg, posturography) may be more sensitive than clinical tests in people with mild stroke to detect limitations in their balance capacity—and changes therein due to motor recovery or following intervention.^17^ With respect to the tests of gait capacity, significant group differences were found for TUG and 10MWT. Yet, the MIC was only reached for group differences in walking speed (0.18 km/hour) as derived from the 10MWT.^50–52^ In line with current recommendations on standardized measurements in people after stroke, the 10MWT therefore also appears to be a useful tool to assess gait capacity in people after mild stroke.^53^

### Clinical Implications

This study highlights the potential impact of a mild stroke on balance and gait capacity, balance confidence fall risk, and daily life physical activity. Decreased balance and gait capacity were found on all clinical tests, that is, the mini-BEST total score and all its sub-domains, TUG, and 10-MWT. An important finding of our study is that even the subgroup of full recoverers demonstrated lower balance and gait capacity compared to healthy control participants. This indicates that our results were not merely driven by individuals with residual—clinically identifiable—motor function impairments of the leg. Our findings therefore suggest that balance and gait capacity after mild stroke may be partly) determined by subtle, clinically unrecognizable, leg motor impairments or perhaps higher-level sensorimotor integration deficits.

Balance and gait limitations are key risk factors for falling.^8^ Given the double fall rate in our mild stroke population, the balance and gait limitations observed in this study appear to be clinically relevant. Hence, individuals after a mild stroke should be considered to receive training aimed at improving balance and/or gait capacity, even in the absence of motor function deficits. Improvements in balance capacity are particularly achieved by challenging and task-specific balance training, functional weight-shifting and/or gait training.^15^ So-called perturbation-based balance training, in which the dynamic balance responses to external perturbations are being practiced could potentially be beneficial to optimize balance capacity in both mild and moderately affected individuals after stroke.^54^

## Supplemental Material

sj-docx-1-nnr-10.1177_15459683231207360 – Supplemental material for Mild Stroke, Serious Problems: Limitations in Balance and Gait Capacity and the Impact on Fall Rate, and Physical Activity Click here for additional data file. Supplemental material, sj-docx-1-nnr-10.1177_15459683231207360 for Mild Stroke, Serious Problems: Limitations in Balance and Gait Capacity and the Impact on Fall Rate, and Physical Activity by Jolanda M. B. Roelofs, Sarah B. Zandvliet, Ingrid M. Schut, Anouk C. M. Huisinga, Alfred C. Schouten, Henk T. Hendricks, Digna de Kam, Leo A. M. Aerden, Johannes B. J. Bussmann, Alexander C. H. Geurts and Vivian Weerdesteyn in Neurorehabilitation and Neural Repair
