# Supplementary material for: Mild Stroke, Serious Problems: Limitations in Balance and Gait Capacity and the Impact on Fall Rate, and Physical Activity
Source: Neurorehabil Neural Repair. 2023 Oct 25;37(11-12):786–98. doi: 10.1177/15459683231207360 (PMC10685695; doi:10.1177/15459683231207360)
Supplement: sj-docx-1-nnr-10.1177_15459683231207360 – Supplemental material for Mild Stroke, Serious Problems: Limitations in Balance and Gait Capacity and the Impact on Fall Rate, and Physical Activity [file sj-docx-1-nnr-10.1177_15459683231207360.docx]

**Supplementary table 1, results of uncorrected analyses**

|  | All participants after mild stroke (n=70) | Full recoverers after mild stroke (n=38)^a^ | Healthy control participants (n=47) | Healthy versus all stroke participants | Healthy versus full recoverers |
| --- | --- | --- | --- | --- | --- |
| *mini-BEST*  *median in points (IQR)* | 24  (4) | 25  (2) | 26  (2) | F(1,114)=23.20,  B:-2.16, CI:-3.04- -1.27, p<0.01 | F(1,82)=7.05,  B:-1.21, CI:-2.12- -0.3 p=0.01 |
| *Timed Up & Go test*  *Mean in seconds (IQR)* | 10.20s  (2.55) | 9.53s  (1.67) | 8.54s  (1.77) | F(1,114)25.31=,  B:1.66, CI:1.01-2.31, p<0.01 | F(1,82)=13.08,  B:0.99, CI:0.45-1.54, p<0.01 |
| *10 meter walk test*  *Mean in kilometer per hour (IQR)* | 4.66 km/h (1.16) | 4.86 km/h  (0.96) | 5.22 km/h  (0.75) | F(1,113)=17.86,  B:, CI:-0.84 - -0.30, p<0.01 | F(1,82)=8.55,  B:-3.67, CI:-0.62- -0.18, p<0.01 |
| Total duration of physical activity  *Mean in minutes per day (IQR)* | 169 min/day  (79) | 180 min/day  (66) | 178 min/day  (65) | F(1,104)=0.86,  B:-9.5, CI:-29.9– 10.8, p=0.36 | F(1,75)=0.04,  B:2.14, CI:-19.18-23.47,  p=0.84 |
| Total duration of walking  *Mean in minutes per day (IQR)* | 150 min/day  (59) | 161 min/day  (204) | 153 min/day  (62) | F(1,104)=0.10,  B:-2.93, CI:-21.54- 15.69, p=0.97 | F(1,75)=0.71,  B:8.46, CI:-11.60-28.51, p=0.40 |
| Total intensity of physical activity  *Mean in counts per minute (IQR)* | 1494 c/min  255 | 1540 c/min  (276) | 1609 c/min  (236) | F(1,104)=7.33,  B:-115, CI:-199 - -31, p=0.08 | F(1,75)=,  B:-69, CI:-173-35,  p=0.19 |
| Total intensity of walking  *Mean in counts per minute (IQR)* | 1439 c/min  (250) | 1472 c/min  (272) | 1520 c/min  (196) | F(1,104)=5.67,  B:-8, CI:-149- -13, p=0.019 | F(1,75)=1.46,  B:-48, CI:-127-31, p=0.23 |

Results of the uncorrected statistical analyses conducted between mild stroke participants, healthy controls as well as a sub-analysis in which full recoverers were compared to healthy control participants.

^a^ Subgroup of mild stroke participants with complete motor recovery of the paretic leg (i.e., Fugl-Meyer Assessment – Lower Extremity =28 and Motricity Index – Lower Extremity =100).
